# Simultaneous Avascular Necrosis of the Lunate and Scaphoid

**Published:** 2017-02-08

**Authors:** Benson J. Pulikkottil, Edward Ruane, Michael E. Scott, Alexandre Philipp Sater, Joseph E. Imbriglia

**Affiliations:** Department of Orthopedic Surgery, University of Pittsburgh Medical Center, Pittsburgh, Pa

**Keywords:** avascular necrosis, lunate, scaphoid, Kienböck's disease, Preiser's disease

## DESCRIPTION

Avascular necrosis (AVN) involving more than 1 carpal bone is a rare entity. In this case, a 60-year-old female smoker with simultaneous AVN of both the scaphoid and the lunate was treated with proximal row carpectomy. Postoperatively, excellent pain relief and increased wrist range of motion were achieved.

## QUESTIONS

1. **What are the theorized etiologies for simultaneous AVN of the lunate and scaphoid?**
2. **Describe the history and physical examination findings associated with this particular patient to aid in diagnosing this rare problem?**
3. **What are the previous treatment regimens described in the literature for this problem?**
4. **On the basis of the current body of literature, how was the treatment regimen optimized for this particular patient?**

## DISCUSSION

Individually, Kienböck's disease and Preiser's disease are described in the literature with a multitude of potential treatment options. Simultaneous AVN of these carpal bones is a rare entity. We have found 4 previous reports of concomitant scaphoid and lunate osteonecrosis. The potential causes mentioned in the literature include trauma, steroids, gout, alcoholism, infections, hyperbaric events, storage disorders, hepatic dysfunction, and autoimmune diseases.^1^^-^3 To date, most cases have been associated with steroid usage; steroid use appears to be total-dose dependent as well as daily-dose dependent.^1^ After traumatic causes,^4^ steroid use is the second most common etiology of AVN.^5^ In addition to steroid usage, smoking confers a 3.0 to 3.9 relative risk for AVN.^6^ Our patient had exposure to both of these potentially causative agents.

A 60-year-old retired right-hand–dominant woman presented with a chief complaint of right-sided dorsocentral wrist pain for a period of approximately 1 year. She rated her pain as an 8 on a scale of 0 to ten. No reported trauma had occurred. She had no systemic illnesses or significant medical history. Her pain was tolerable initially, but over the past few months, it had worsened and she was unable to perform her daily activities without severe pain. She reported smoking 1 pack of cigarettes per day for more than 20 years. She was initially seen at an outside hospital twice prior to our encounter. Both times she was treated conservatively with only immobilization, nonsteroidal anti-inflammatory medications, and approximately a month of systemic steroids (prednisone). On examination, she had an effusion with swelling over the radiocarpal joint. She had pain with resisted wrist flexion and extension. Her right wrist had an active 5° extension and 10° flexion.

Budoff,^1^ Park et al,^3^ and Bhardwaj et al^2^ provided a basis for this rare problem: Budoff^1^ described a 50-year-old left-handed woman with prior steroid usage and bilateral dorsocentral wrist pain. Ultimately, she was noted to have right-sided scaphoid and lunate AVN and was treated with proximal row carpectomy. Despite postoperative radiocapitate arthritis, her pain and range of movement significantly improved. Park et al^3^ described a 56-year-old woman with a 20-year history of right wrist pain and no prior steroid use. She developed scaphoid and lunate AVN and underwent radioscapholunate fusion. She reported improved range of movement and is currently pain-free.^3^ Bhardwaj et al^2^ reported of a 20-year-old man with a 3-month history of right wrist pain. He had been taking herbal medications and steroids. He presented with scaphoid and lunate AVN and underwent proximal row carpectomy. Similar to the aforementioned reports, he returned to his normal activities with adequate relief of pain.^2^

Our patient fits the mold of prior reported instances of scaphoid and lunate AVN. She used steroids and was an avid smoker. A discernable difference was her very rapid worsening of symptoms and rapid changes on radiographs. She accepted the risks of potential arthritic capitate and lunate surfaces with proximal row carpectomy and radial styloidectomy. Fortunately, her cartilage surfaces were intact. Our only other option was a complete wrist fusion, which she did not want to pursue. Postoperatively, she had excellent pain relief and increased range of motion of the wrist. By just 4 months postoperatively, she showed great improvement in wrist function with active 25° extension and 40° flexion. This represents a 20° improvement in extension and 30° improvement in flexion She understands that there is the potential for her to develop radiocapitate arthritis.^7^ If in the future she develops intolerable pain, she may need to undergo a wrist arthrodesis.

## Figures and Tables

**Figure 1:**
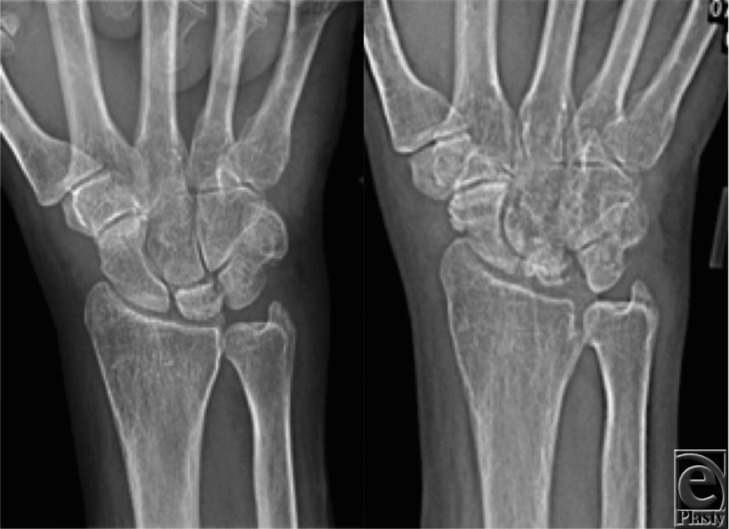
Preoperative posteroanterior radiographs showing progressive fragmentation and collapse of the lunate, as well as subsequent pathologic fracture of the scaphoid.

**Figure 2:**
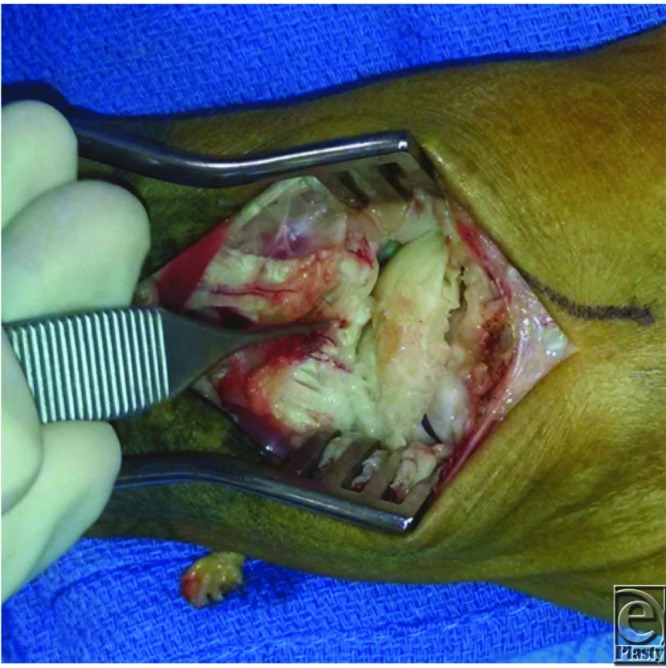
Intraoperative photograph showing the fragmented and avascular appearance of the scaphoid and lunate.

**Figure 3:**
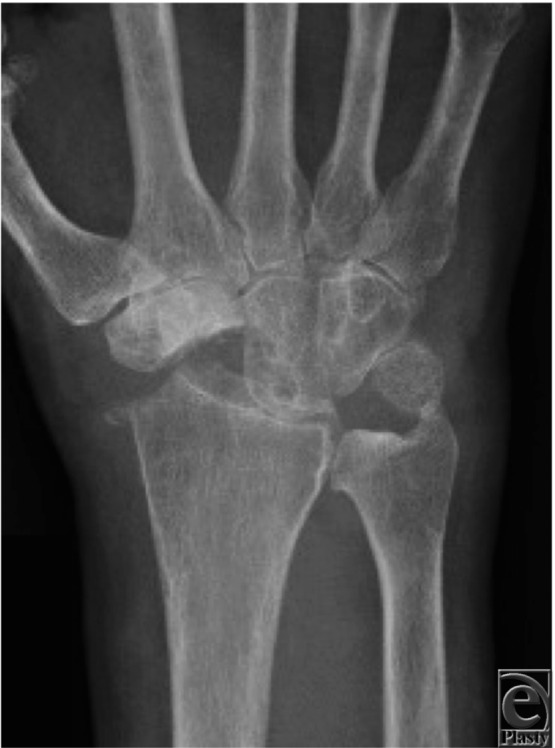
Postoperative posteroanterior radiograph showing satisfactory positioning of the capitate in the lunate facet.
